# A new and efficient synthesis of substituted 2-amino-1,3,4-oxadiazoles from isothiocyanates and hydrazides

**DOI:** 10.1186/s13065-026-01845-7

**Published:** 2026-06-21

**Authors:** Lei Wu, Haiyign Tian, Yuying Zhang, Wenjing Ma, Qi Zhang, Ruiling Chen, Xiuling Chen

**Affiliations:** 1 Institute of New Carbon-Based Materials and Zero-Carbon and Negative-Carbon Technology, Lyuliang University, Lvliang, 033001 China; 2 School of Pharmacy, Changzhi Medical College, Changzhi, 046000 China; 3 School of Chemical Engineering, Guangdong University of Petrochemical Technology, Maoming, 525000 China; 4 School of Pharmacy, Hubei University of Science and Technology, Xianning, 437100 China

**Keywords:** 2-Amino-1,3,4-oxadiazoles, Metal-free, Isothiocyanates, Hydrazides

## Abstract

1,3,4-Oxadiazoles are privileged heterocyclic scaffolds with broad bioactivities, holding significant importance in pharmaceutical and agrochemical discovery. Herein, we report a novel, efficient, and simple synthetic route to 2-amino-1,3,4-oxadiazoles via NaOH-mediated desulfurative cyclization of hydrazides and isothiocyanates under mild reaction conditions. This developed methodology features broad substrate tolerance, excellent yields, operational simplicity, and mild conditions, providing a straightforward pathway for versatile syntheses of valuable 1,3,4-oxadiazole derivative. Consequently, the present reaction opens an alternative path for the preparation of 1,3,4-oxadiazoles via regioselective cyclization hydrazides and isothiocyanates.

**Supplementary Information:**

The online version contains supplementary material available at https://doi.org/10.1186/s13065-026-01845-7.

## Introduction

Nitrogen-containing heterocycles are fundamental structural motifs in pharmaceutical and agrochemical research due to their diverse biological activities [1]. Among these heterocyclic frameworks, five-membered ring systems serve as the pivotal cores of a large number of bioactive compounds. In particular, the 1,3,4-oxadiazole skeleton has emerged as an invaluable scaffold for drug discovery [2, 3]. It a privileged structure in modern medicinal chemistry, exhibiting excellent resistance to metabolic and hydrolytic degradation, avorable physicochemical and pharmacokinetic properties [4]. The pharmacological potency of 1,3,4-oxadiazole derivatives can be enhanced via specific hydrogen-bond interactions with biological targets. These compounds possess a wide spectrum of pharmacological properties, including antitumor, anticonvulsant, anti-inflammatory, antimicrobial and antiviral activities. [5–9]. This privileged scaffold is present in multiple clinically approved pharmaceuticals, such as the antibacterial furazidin, the antihypertensive nesapidil, the metabolic modulator AZD3988, and the antiviral agent raltegravir (Isentress®) [10, 11]. In addition to their biomedical applications, 1,3,4-oxadiazoles also exhibit promising potential in materials science, with widespread utilization in organic light-emitting diodes (OLEDs), gas separation membranes [12, 13], and anticorrosive coatings.

**Scheme 1:**
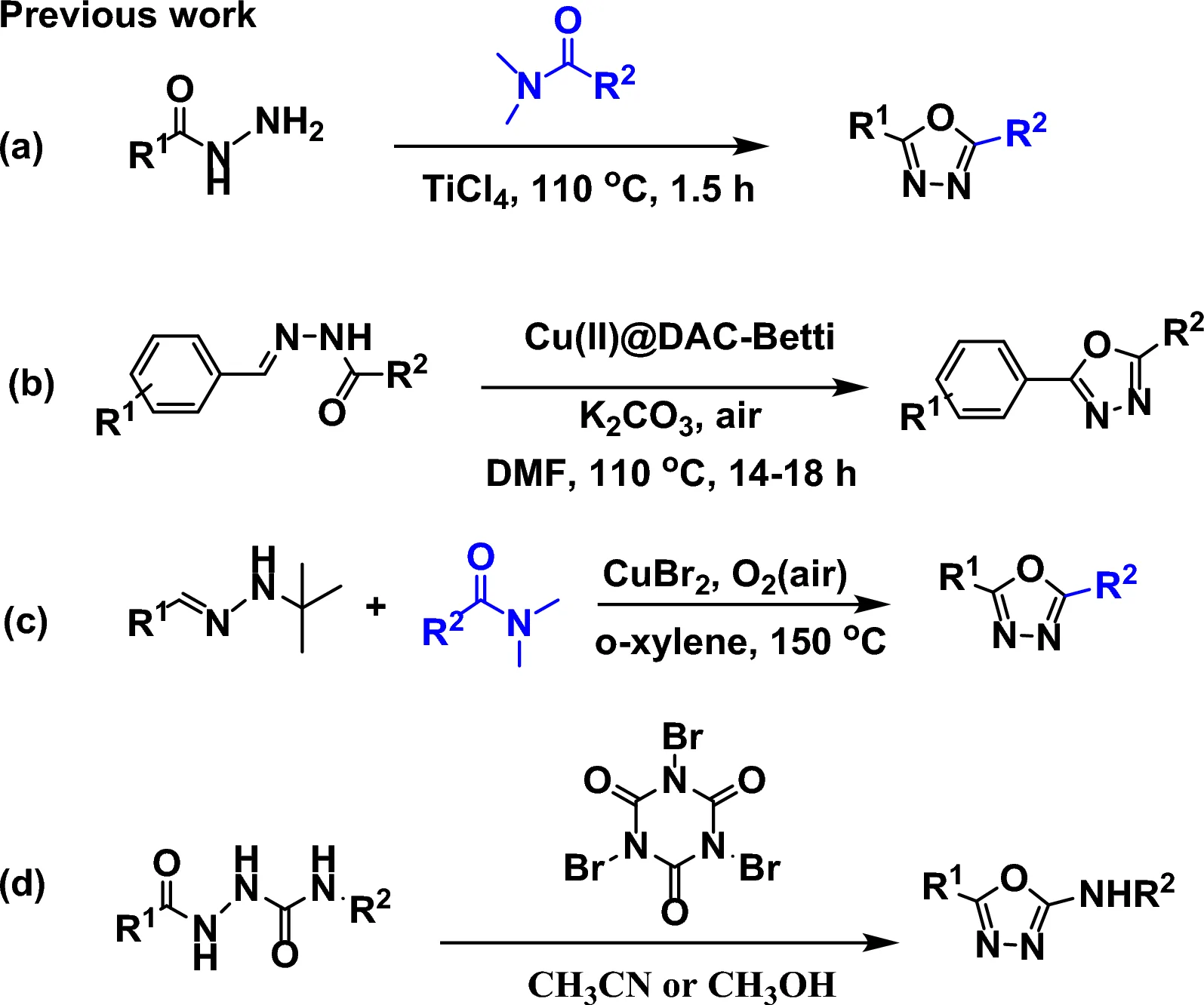
The previous route to synthesis 1,3,4-oxadiazole

The widespread applicability of 2-amino-1,3,4-oxadiazoles has driven the development of efficient synthetic methodologies [14–18]. A commonly employed two-step synthetic route consists: (1) the condensation of hydrazides with isothiocyanates to yield thiosemicarbazides, (2) cyclodehydration coupled with desulfurization to construct the corresponding heterocyclic skeleton [19]. Among the reported one-step synthetic strategies, the 1,3,4-oxadiazole ring is most commonly constructed via the dehydrative cyclization of (di)acylhydrazines or the oxidative cyclization of acylhydrazones. Furthermore, 2-amino-1,3,4-oxadiazole derivatives can also be synthesized through cyclodehydrosulfurization with the aid of various oxidants, such as KHSO_4_, I_2_/K_2_CO_3_, o-iodoxybenzoic acid (IBX), O-(benzotriazol-1-yl)-*N,N,N',N'*-tetramethyluronium tetrafluoroborate (TBTU), and CuI [20–24]. Zhang and coworkers developed a solvent-free protocol mediated by TiCl_4_ for transamidation and cyclization. Nevertheless, the strong corrosivity of TiCl_4_ inevitably brings about severe safety hazards (Scheme 1a) [25]. Fereshteh Bahri et al. employed Cu(II) species to form a six-membered metallacyclic intermediate, which undergoes reductive elimination to afford the target product. However, this synthetic strategy suffers from the drawbacks of high reaction temperature and prolonged reaction time (Scheme 1b) [26]. Liu and Feng established a Cu(II)-mediated aerobic oxidation of hydrazones proceeding through coordination, cyclization, and aromatization sequences, although the substrate compatibility of this method remains constrained (Scheme 1c) [27]. Mattos et al. employed TBCA as a reagent for the cyclodehydrosulfurization of 1-acylthiosemicarbazides to access substituted 2-amino-1,3,4-oxadiazoles (Scheme 1d) [28]. Although these existing methodologies enable efficient synthesis of 2-amino-1,3,4-oxadiazole derivatives. They generally depend on specialized substrates, harsh conditions, and stoichiometric oxidants, which restrict their practical applicability.

**Scheme 2:**
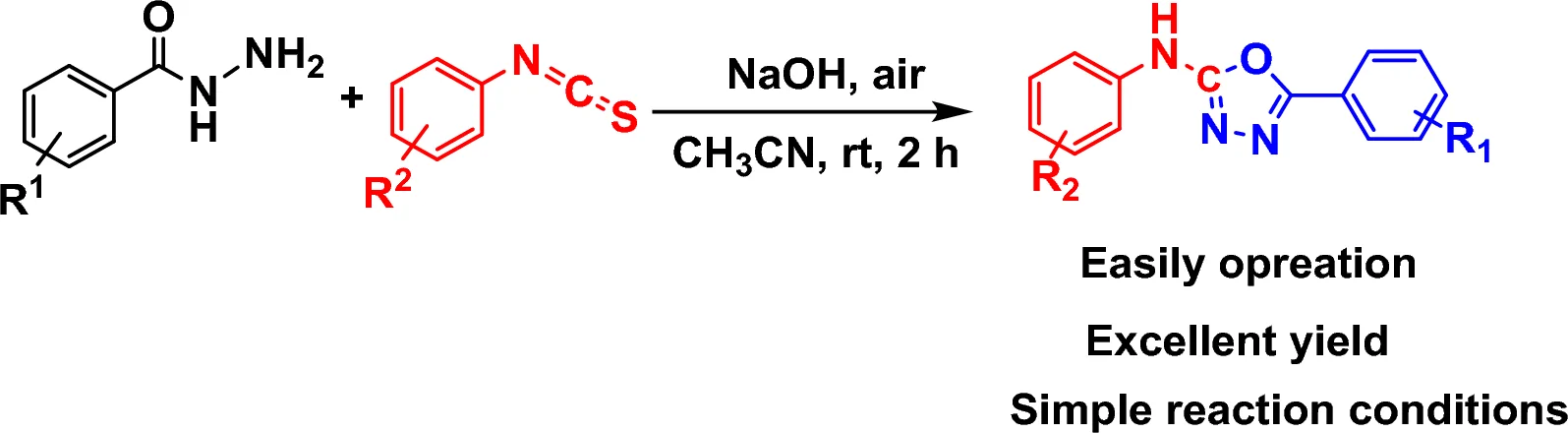
The route of 1,3,4-oxadiazole synthesis

Herein, we disclose a highly efficient, facile, and practical method for the construction of 1,3,4-oxadiazole derivatives under mild and operationally simple reaction conditions (Scheme 2). This optimized synthetic protocol utilizes readily available substituted isothiocyanates and hydrazides as the starting precursors, with inexpensive sodium hydroxide serving as an efficient promoter in acetonitrile as the reaction medium. This synthetic protocol features simple operational procedures and remarkable functional group compatibility. Notably, aromatic isothiocyanates bearing either electron-donating or electron-withdrawing substituents are well tolerated in this transformation. Meanwhile, aliphatic substrates also smoothly participate in the reaction, delivering the corresponding 1,3,4-oxadiazole derivatives with good to excellent yields. Consequently, this protocol provides an alternative synthetic route toward 1,3,4-oxadiazoles through the regioselective cyclization of hydrazides with isothiocyanates.

To identify suitable reaction conditions for synthesizing substituted 1,3,4-oxadiazoles from benzoic hydrazide and phenyl isothiocyanate, initial optimization was performed using **1a** and **2a** as model substrates in the presence of a base, the results are summarized in Table 1. Gratifyingly, **3a** (N,5-diphenyl-1,3,4-oxadiazol-2-amine) was obtained in 26% yield with Et_3_N (Table 1, entry 1). Further screening of different bases (Table 1, entries 2–6) demonstrated that sodium hydroxide (NaOH) delivered the best performance, furnishing target product **3a** in a satisfactory yield of 74% (Table 1, entry 6). Subsequent solvent screening investigations indicated that most of the examined media, including toluene, chlorobenzene, 1,4-dioxane, DCE, DMF, DMSO, isopropanol and methanol, failed to further improve the product yield of this transformation (Table 1, entries 7–12). The **3a** compound was not obtained in the absence of NaOH, which demonstrated that the base played a key role in this transformation (Table 1, entry 13). Next the effect of temperature on the product formation was also studied subsequently. It was observed that the yield of **3a** was no significant change when the reaction temperature was increased to 50 °C to120 ^o^C (Table 1, entries 14–17). Thus, the optimized conditions for the synthesis of 1,3,4-oxadiazoles **3a** are as follows: benzo hydrazides **1a** (0.2 mmol), phenyl isothiocyanates **2a** (0.3 mmol), NaOH (0.4 mmol), CH_3_CN (3 mL) at 25 °C for 2 h.

**Table 1:** Optimization of reaction conditions^a^

**Figure:**
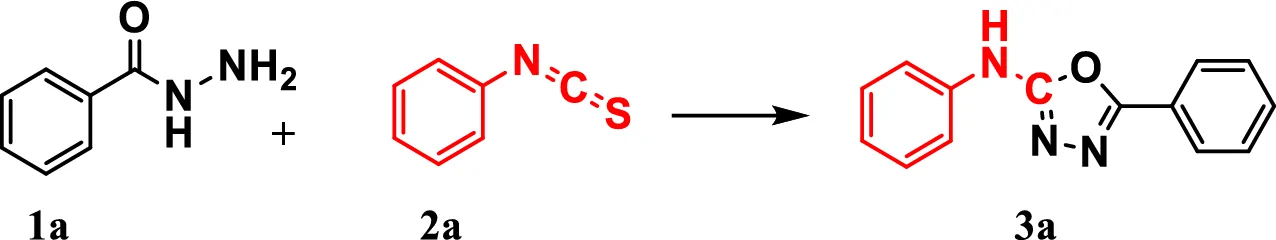

Entry	Base	Solvent	3a%^b^
1	Et_3_N	CH_3_CN	26%
2	KOH	CH_3_CN	56%
3	DBU	CH_3_CN	44%
4	K₂CO₃	CH_3_CN	31%
5	Cs₂CO₃	CH_3_CN	65%
6	NaOH	CH_3_CN	76%
7	NaOH	Toluene	10%
8	NaOH	chlorobenzene	65%
9	NaOH	DCE	60%
10	NaOH	1,4-Dioxane	40%
11	NaOH	DMF	15%
12	NaOH	DMSO	15%
13	–	CH_3_CN	trace
14^*c*^	NaOH	CH_3_CN	70%
15^d^	NaOH	CH_3_CN	72%
16^e^	NaOH	CH_3_CN	70%
17^*f*^	NaOH	CH_3_CN	69%

^a^Reaction conditions: benzo hydrazide 1a (0.2 mmol), phenyl isothiocyanate 2a (0.3 mmol), bases (0.4 mmol), solvents (3 mL), 25 mL Schlenk tube, air, 25 °C, 2 h. ^b^Isolated yield. ^*c*^50 ^o^C, ^*c*^80 ^o^C, ^*c*^100 ^o^C, ^*f*^120 ^o^C.

Under the established optimal reaction conditions, a broad spectrum of substituted hydrazides (**1a–1n**) were efficiently reacted with phenyl isothiocyanate (**2a**). As summarized in Table 2, these hydrazide derivatives exhibited good compatibility with the current transformation, demonstrating that the NaOH-promoted desulfurization and cyclization protocol is generally applicable for the facile synthesis of diverse 2-amino-1,3,4-oxadiazoles (**3a–3n**) with good to excellent yields. To begin with, unsubstituted benzoyl hydrazide as well as bromo- and chloro-substituted benzoyl hydrazides were examined as representative substrates under standard conditions, delivering the corresponding products **3a–3c** in excellent yields (Table 2, entries 1–3). Notably, even the strongly electron-withdrawing nitro substituent in 4-nitrobenzoyl hydrazide (**1d**) was well tolerated in this system, affording the target product **3d** in 74% yield (Table 2, entry 4). By contrast, hydrazides functionalized with electron-donating groups (–CH_3_, –OCH_3_, –OH, –NH_2_) exhibited higher reactivity, furnishing the corresponding 2-amino-1,3,4-oxadiazoles **3e–3 h** in moderate to excellent yields of 77–88% (Table 2, entries 5–8). In general, the yield trend indicated that electron-donating substituents on benzoyl hydrazides could more effectively facilitate the reaction compared with electron-withdrawing counterparts. Heteroaromatic carbohydrazides, including 2-pyridinecarbohydrazide (**1i**), 2-thiophenecarbohydrazide (**1j**), and 3,4-methylenedioxybenzoyl hydrazide (**1 k**), also proceeded smoothly under the standard conditions to afford the corresponding 2-amino-1,3,4-oxadiazole derivatives **3i–3 k** in good yields. This result further demonstrates the excellent compatibility of this protocol toward heterocyclic structural motifs (Table 2, entries 9–11). Moreover, 2-naphthoyl hydrazide (**1 l**), as well as aliphatic hydrazide substrates including butyryl hydrazide (**1 m**) and phenylacetyl hydrazide (**1n**), efficiently delivered the target products **3 l–3n** in good to high yields (Table 2, entries 12–14). These results firmly validate the broad substrate scope and general applicability of the developed synthetic strategy.

**Table 2:**
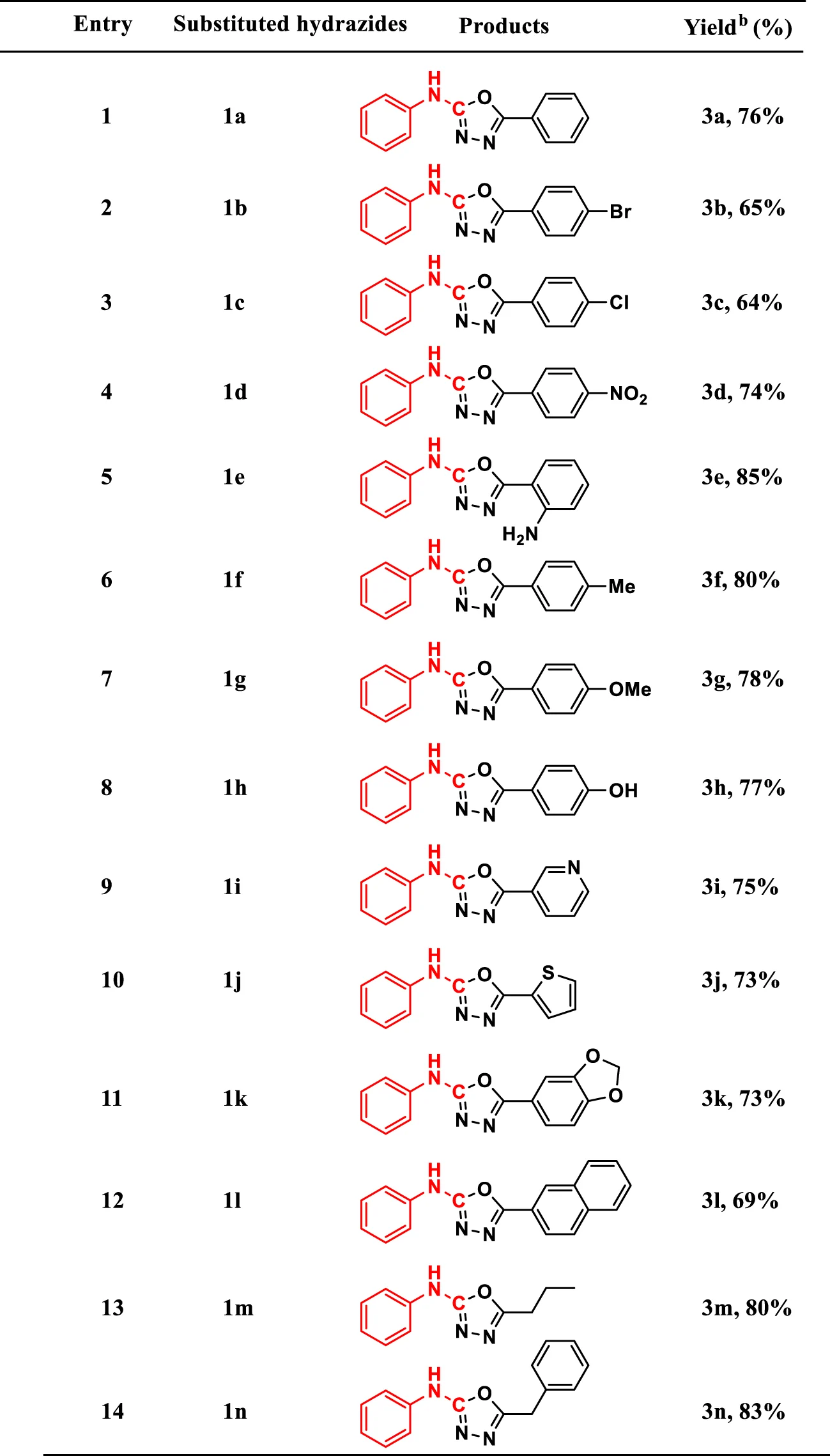
Hydrazide substrates with varying substituents^a^

**Figure:**
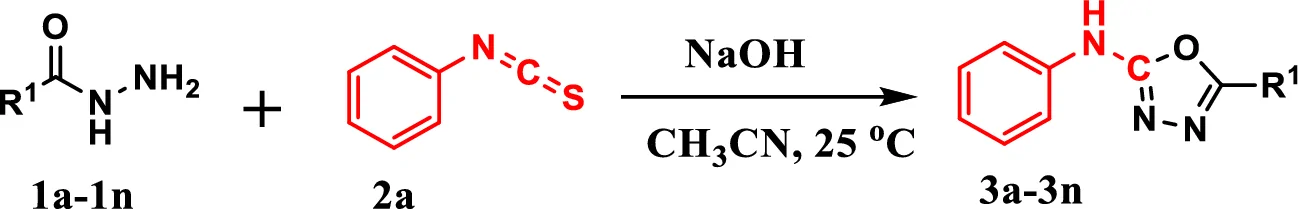

^a^Reaction conditions: substituted hydrazides 1a-1n (0.2 mmol), phenyl isothiocyanate 2a (0.3 mmol), NaOH (0.4 mmol), CH_3_CN (3 ml), in 25 mL Schlenk tube, 25 °C, 2 h

^b^Isolated yield.

The scope of substituted isothiocyanates was examined using benzoyl hydrazide under the optimized reaction conditions. As summarized in Table 3, a broad range of substituted isothiocyanates were tolerated in this transformation for the synthesis of 2-amino-1,3,4-oxadiazoles. The reaction efficiency remained unaffected by variations in the position or electronic nature of the substituents. Notably, aromatic isothiocyanates bearing electron-donating groups—including *p*-chlorophenyl (**2b**), *p*-bromophenyl (**2c**), *p*-methylphenyl (**2d**), and *p*-methoxyphenyl (**2e**)—furnished products **3o–3r** in good yields (Table 3, entries 2–5). Similarly, isothiocyanates bearing electron-withdrawing substituents, such as *p*-nitrophenyl isothiocyanate (**2f**) and *p*-(trifluoromethyl)phenyl isothiocyanate (**2 g**), reacted smoothly with benzoyl hydrazide under the standard conditions, affording the desired products **3 s** and **3t** in 75% and 66% yields, respectively (Table 3, entries 6–7). The substituent electronic effect of isothiocyanate substrates was also investigated. The results indicated that electron-donating substituents are more favorable for promoting the reaction process, which is consistent with previous literature reports [2]. Furthermore, the present protocol exhibits good compatibility toward both aliphatic and benzylic isothiocyanates. For instance, benzyl isothiocyanate (**2 h**) reacted smoothly with benzoyl hydrazide to afford the corresponding product 3u in 82% yield (Table 3, entry 8). Notably, aliphatic isothiocyanates, including propyl (**2i**) and butyl (**2j**) analogues, were also well tolerated in this transformation, furnishing the corresponding products **3v** and **3w** in 82% and 80% yields, respectively (Table 3, entries 9–10). In addition, ortho-substituted phenyl isothiocyanate (**2 k**) successfully participated in the reaction to give product **3 × **in 64% yield (Table 3, entry 11). This result reveals that the reaction is insensitive to steric hindrance arising from ortho-substitution on the aromatic ring.

**Table 3:**
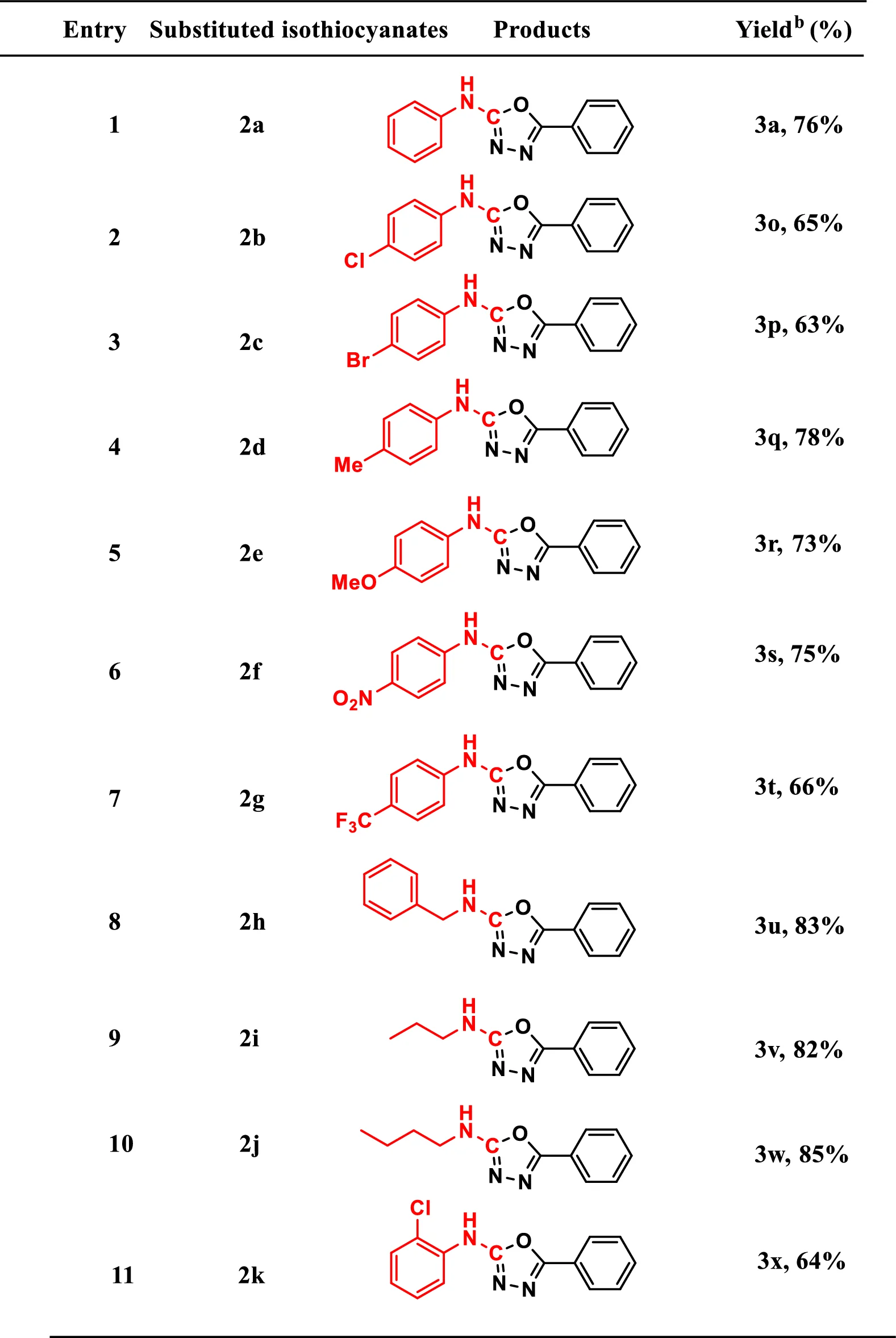
Hydrazide substrates with varying substituents^a^

**Figure:**
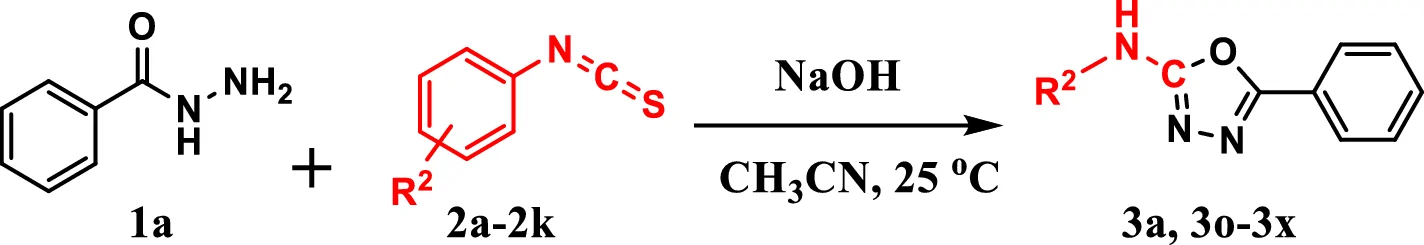

^a^Reaction conditions: benzoyl hydrazine 1a (0.2 mmol), substituted isothiocyanates 2a-2j (0.3 mmol), NaOH (0.4 mmol), CH_3_CN (3 ml), in 25 mL Schlenk tube, 25 °C, 2 h

^b^Isolated yield.

Overall, both substituted benzoyl hydrazides and phenyl isothiocyanates are well compatible with electron-withdrawing and electron-donating functional groups, heterocyclic skeletons, and substrates with varied steric environments. Under the optimal conditions, these substrates can be efficiently converted into the corresponding substituted 2-amino-1,3,4-oxadiazole derivatives. Collectively, these results further verify that the present transformation features operational simplicity and excellent functional group tolerance.

**Scheme 3:**
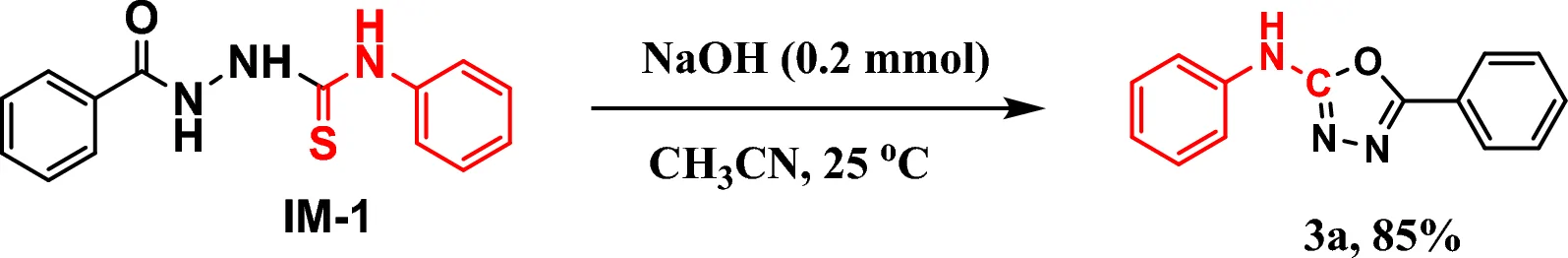
Controlled experiment

To gain insight into the plausible reaction mechanism, a series of control experiments were carried out. The reaction of benzohydrazide with phenyl isothiocyanate in the presence of triethylamine at room temperature successfully afforded the thiosemicarbazide intermediate **IM-1**. Subsequently, when **IM-1** was employed directly as the starting material, the target product **3a** was isolated in 85% yield. Moreover, only a stoichiometric amount of NaOH (0.2 mmol) was required under the optimal conditions, further verifying that the reaction proceeds preferentially through the formation of intermediate **IM-1** (Scheme 3).

**Scheme 4:**
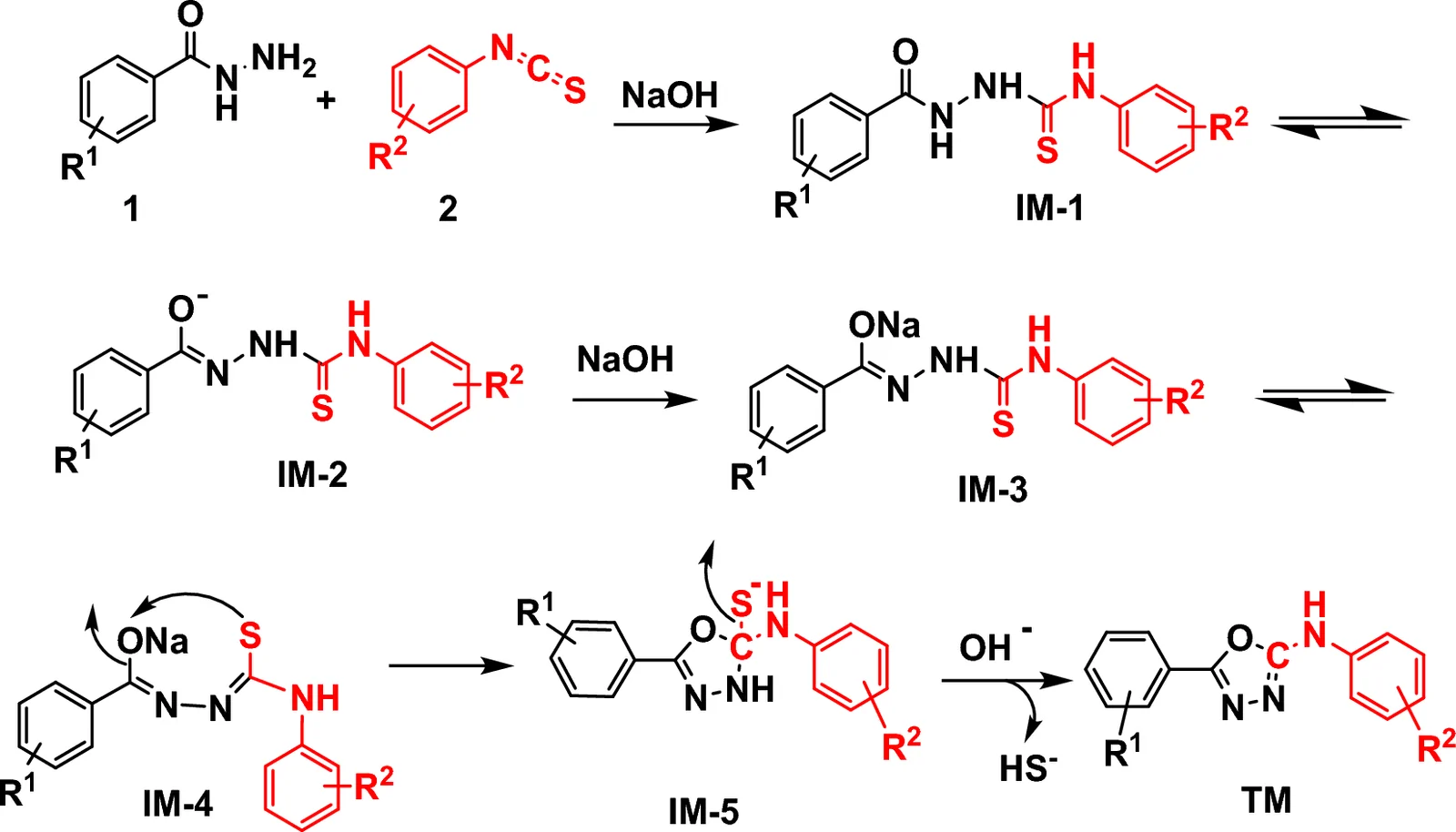
Plausible reaction pathway for the synthesis of 2-amino-1,3,4-oxadiazoles

Based on the above control experimental results and previous literature precedents [2, 28–31], a plausible NaOH-promoted desulfurization–cyclization mechanism for the construction of 2-amino-1,3,4-oxadiazoles from hydrazides and isothiocyanates is proposed, as depicted in Scheme 4. Initially, benzohydrazide condenses with phenyl isothiocyanate to generate the 1-acylthiosemicarbazide intermediate IM-1, and this condensation process can proceed even in the absence of NaOH [2]. Intermediate **IM-1** first undergoes amide tautomerization under basic conditions to form **IM-2**. Subsequently, the resulting oxyanion coordinates with sodium cation to furnish intermediate **IM-3**, which then undergoes intramolecular nucleophilic attack by the sulfur atom to yield **IM-4**. Thereafter, **IM-4** proceeds via intramolecular ring closure to generate cyclic intermediate **IM-5**. Finally, further treatment with NaOH promotes aromatization through the elimination of HS^−^, ultimately affording the target 2-amino-1,3,4-oxadiazole product **TM**.

## Conclusions

In summary, we have developed a green and efficient protocol for the synthesis of 2-amino-1,3,4-oxadiazole derivatives using readily available substituted isothiocyanates and hydrazides under mild reaction conditions. This synthetic method features a broad substrate scope, excellent product yields, operational simplicity, and mild reaction parameters, thus providing a practical and facile route to diverse 2-amino-1,3,4-oxadiazole scaffolds. Furthermore, this work establishes an effective strategy for the regioselective cyclization of hydrazides with isothiocyanates via a straightforward experimental procedure, and offers valuable insights for the rational design and large-scale preparation of functionalized 1,3,4-oxadiazole derivatives.

## Supplementary Information


Supplementary Material 1.


## Data Availability

The data sets used and analysed during the current study are available from the corresponding author on reasonable request. We have presented all data in the form of Tables and Figures in the manuscript and support information.
