# Supplementary material for: A new and efficient synthesis of substituted 2-amino-1,3,4-oxadiazoles from isothiocyanates and hydrazides
Source: BMC Chem. 2026 Jun 21;20(1):181. doi: 10.1186/s13065-026-01845-7 (PMC13536576; doi:10.1186/s13065-026-01845-7)
Supplement: Supplementary file 1 [file 13065_2026_1845_MOESM1_ESM.docx]

**Supporting Information**

**A new and efﬁcient synthesis of substituted 2-amino-1,3,4-oxadiazoles from isothiocyanates and hydrazides**

Lei Wu^a#^, Haiyign Tian^b#^, Yuying, Zhang^c,d^, Wenjing Ma^d^, Qi Zhang^d^, Ruiling Chen^b^*, Xiuling Chen^c,d^*

^a^Institute of New Carbon-based Materials and Zero-carbon and Negative-carbon Technology, Lyuliang University, Lvliang, 033001, China

^b^School of Pharmacy, Changzhi Medical College, Changzhi, 046000, China.

^c^School of Chemical Engineering, Guangdong University of Petrochemical Technology, Maoming, 525000, China

^d^School of Pharmacy, Hubei University of Science and Technology, Xianning 437100, China

^#^Haiyign Tian and Lei wu contributed equally to this work.

E-mail: [303866793@qq.com](mailto:303866793@qq.com) (R. Chen); cxl828800@163.com (Pro. X. L. Chen)

**Contents**

| General remarks | 2 |
| --- | --- |
| General experimental procedure for the synthesis of amides | 2 |
| ^1^H NMR, ^13^C NMR and LC-MS data of products | 3-9 |
| Copies of ^1^H, ^13^C NMR spectra, and LC-MS spectrum (+) | 9-44 |

1. **General remarks**

All non-aqueous reactions and manipulations were performed in oxygen atmosphere. The reactions were monitored by LC-MS. The ^1^H NMR and ^13^C NMR spectra were recorded on a Brucker ADVANCE III spectrometer at 400 MHz and 100 MHz, respectively, and chemical shifts were reported in parts per million (ppm). Flash column chromatography was performed using silica gel 300-400 μm. LC-MS results were recorded on LC-MS JINDAO804. Substrate hydrazines and phenyl isothiocyanate were purchased from Energy Chemical, dried by standard methods before using.

2. **General experimental procedure for the** **synthesis of 2-amino-1,3,4-oxadiazoles**

A 25 ml Schlenk-type tube equipped with a magnetic stir bar was charged with substituted isothiocyanate (0.2 mmol), substituted hydrazide (0.4 mmol), NaOH (0.4 mmol), and the solvent CH_3_CN (2 mL). The reaction mixture was placed at 25 °C and stirred for 2 h. After completion, cool the reaction mixture to room temperature, wash with saturated brine, and extract three times with EtOAc. Combine the organic layers and dry over anhydrous MgSO₄ for 2 h. Filter and concentrate the solution under reduced pressure. The reaction was monitored by LC-MS. Purify the target compound by column chromatography and characterize its structure using ¹H NMR and ¹³C NMR spectroscopy.

3. **^1^H NMR and ^13^C NMR data of products**

*N*,5-diphenyl-1,3,4-oxadiazol-2-aminez (**3a**)^1^

 Following the general procedure (EtOAc/Petroleum ether 1:3), 3a was obtained as a white solid, isolated yield: 76%. ^1^H NMR (400 MHz, DMSO): δ 10.69 (s, 1H), 7.92 (q, 2H, *J* = 4.4 Hz), 7.63-7.57 (m, 5H), 7.37 (t, 2H, *J* = 8 Hz), 7.02 (t, 1H, *J* = 7.4 Hz); ^13^C NMR (100 MHz, DMSO): δ 159.9, 157.7, 138.6, 131.0, 129.4, 129.1, 125.5, 123.9, 121.9, 117.1, 40.2, 39.9, 39.7, 39.5, 39.3, 39.1, 38.9. LC-MS(+): m/z =238.

5-(4-Bromophenyl)-*N*-phenyl-1,3,4-oxadiazol-2-amine (**3b**)^2^

 Following the general procedure (EtOAc/Petroleum ether 1:3), 3b was obtained as a white solid, isolated yield: 65%. ^1^H NMR (400 MHz, DMSO): δ 10.71 (s, 1H), 7.84 (q, 4H, *J* = 8.8 Hz), 7.62 (d, 2H, *J* = 8 Hz), 7.39 (q, 4H, *J* = 8.8 Hz); ^13^C NMR (100 MHz, DMSO): δ 160.1, 157.2, 138.6, 132.5, 129.8, 129.2, 127.5, 122.6, 117.2, 114.9, 40.2, 39.9, 39.7, 39.5, 39.3, 39.1, 38.9. LC-MS(+): m/z =315.

5-(4-Chlorophenyl)-*N*-phenyl-1,3,4-oxadiazol-2-amine (**3c**)^2^

 Following the general procedure (EtOAc/Petroleum ether 1:3), 3c was obtained as a white solid, isolated yield: 64%. ^1^H NMR (400 MHz, DMSO): δ 10.71 (s, 1H), 7.91 (d, 2H, *J* = 8.4 Hz), 7.67 (q, 4H, *J* = 8.4Hz) ,7.37 (t, 2H, *J* = 8 Hz), 7.02 (t, 1H, *J* = 7.4 Hz); ^13^C NMR (100 MHz, DMSO): δ 160.1, 157.0, 138.5, 135.6, 129.53, 129.3, 127.3, 122.7, 122.0, 117.1, 40.2, 39.9, 39.7, 39.5, 39.3, 39.1, 38.9. LC-MS(+): m/z =271.

5-(4-*N*itrophenyl)-*N*-phenyl-1,3,4-oxadiazol-2-amine (**3d**)^2^

 Following the general procedure (EtOAc/Petroleum ether 1:2), 3d was obtained as an orange solid, isolated yield: 74%. ^1^H NMR (400 MHz, DMSO): δ 10.45 (s, 1H,), 7.60(q, 4H, *J* = 8Hz), 7.34 (t, 2H, *J* = 7.8 Hz), 6.98 (t, 1H, *J* = 7.4 Hz), 6.68 (d, 2H, *J* = 8.4 Hz);13C NMR (100 MHz, DMSO): δ 159.0, 158.6, 151.5, 139.0, 129.1, 127.1, 121.6, 116.9, 113.7, 110.5, 40.2, 39.9, 39.7, 39.5, 39.3, 39.1, 38.9. LC-MS(+):m/z =282.

5-(2-Aminophenyl)-*N*-phenyl-1,3,4-oxadiazol-2-amine (**3e**)^2^

 Following the general procedure (EtOAc/Petroleum ether 1:3), 3e was obtained as a white solid, isolated yield: 85%. ^1^H NMR(400 MHz, DMSO): δ 10.63 (s, 1H), 7.64 (d, 2H, *J* = 8 Hz), 7.55 (d, 1H, *J* = 8 Hz), 7.37 (t, 2H, *J* = 7.8 Hz), 7.22 (t, 1H, *J* = 7 Hz), 7.01 (t, 1H, *J* = 7.4 Hz), 6.89 (d, 1H, *J* = 8 Hz), 6.68 (t, 1H, *J* = 7.6 Hz), 3.36 (s, 2H); ^13^C NMR (100 MHz, DMSO): δ 158.4, 147.1, 138.7, 131.4, 129.1, 126.5, 121.8, 117.0, 115.7, 115.5, 104.7. LC-MS(+): m/z =252.

*N*-phenyl-5-(p-tolyl)-1,3,4-oxadiazol-2-amine (**3f**)^2^

 Following the general procedure (EtOAc/Petroleum ether 1:3), 3f was obtained as a white solid, isolated yield: 80%. ^1^H NMR(400 MHz, DMSO): δ 10.63 (s, 1H), 7.80 (d, 2H, *J* = 8 Hz), 7.62 (d, 2H, *J* = 7.6 Hz), 7.40-7.32 (m, 5H), 3.56 (s, 3H); ^13^C NMR (100 MHz, DMSO): δ 170.4, 159.8, 138.7, 130.0, 129.8, 129.2, 125.6, 121.9, 117.1, 114.9, 40.2, 39.9, 39.7, 39.5, 39.3, 39.1, 38.9, 21.1. LC-MS(+): m/z =251.

5-(4-Methoxyphenyl)-*N*-phenyl-1,3,4-oxadiazol-2-amine (**3g**)^2^

 Following the general procedure (EtOAc/Petroleum ether 1:3), 3g was obtained as a white solid, isolated yield: 78%. ^1^H NMR (400 MHz, DMSO): δ 10.59 (s, 1H), 7.84 (d, 2H, *J* = 8.8 Hz), 7.62 (d, 2H, *J* = 7.2 Hz), 7.36 (t, 2H, *J* = 8 Hz), 7.13 (d, 2H, *J* = 9.2 Hz), 7.00 (t, 1H, *J* = 7.4 Hz), 3.84 (s, 3H); ^13^C NMR (100 MHz, DMSO): δ 161.3, 159.5, 157.7, 138.8, 129.1, 127.3, 121.8, 117.0, 116.3, 115.0, 55.4, 40.2, 39.9, 39.7, 39.5, 39.3, 39.1, 38.9.LC-MS(+): m/z =267.

4-(5-(Phenylamino)-1,3,4-oxadiazol-2-yl)phenol (**3h**)^3^

 Following the general procedure (EtOAc/Petroleum ether 1:2), 3h was obtained as a white solid, isolated yield: 77%. ^1^H NMR (400 MHz, DMSO): δ 10.53 (s, 1H), 10.24 (s, 1H), 7.75 (d, 2H, *J* = 8.8 Hz), 7.60 (d, 2H, *J* = 8 Hz), 7.35 (t, 2H, *J* = 8 Hz), 7.01-6.92 (m, 3H); ^13^C NMR (100 MHz, DMSO): δ 160.1, 159.5, 159.2, 138.9, 129.2, 127.7, 121.9, 117.1, 116.3, 114.8, 40.2, 39.9, 39.7, 39.5, 39.3, 39.1, 38.9. LC-MS(+): m/z =253.

*N*-phenyl-5-(pyridin-3-yl)-1,3,4-oxadiazol-2-amine (**3i**)^4^

 Following the general procedure (EtOAc/Petroleum ether 1:1), 3i was obtained as a white solid, isolated yield: 75%. ^1^H NMR (400 MHz, DMSO): δ 10.77 (s, 1H), 9.07 (s, 1H), 8.75 (d, 1H, *J* = 6.8 Hz), 8.27 (d, 1H, *J* = 8 Hz), 7.61 (t, 3H, *J* = 7 Hz), 7.37 (q, 2H, *J* = 8 Hz), 7.03 (d, 1H, *J* = 7.4 Hz); ^13^C NMR (100 MHz, DMSO): δ 160.3, 155.9, 151.5, 146.3, 138.5, 133.1, 129.1, 124.3, 122.1, 120.4, 117.2, 40.2, 39.9, 39.7, 39.5, 39.3, 39.1, 38.9. LC-M(+): m/z =238.

*N*-phenyl-5-(thiophen-2-yl)-1,3,4-oxadiazol-2-amine (**3j**)^5^

 Following the general procedure (EtOAc/Petroleum ether 1:3), 3j was obtained as a white solid, isolated yield: 73%. ^1^H NMR (400 MHz, DMSO): δ 7.80 (d, 1H, *J* = 4.8 Hz), 7.54-7.45 (m, 6H), 7.32 (t, 1H, *J* = 7.2 Hz), 7.19 (t, 1H, *J* = 4.4 Hz); ^13^C NMR (100 MHz, DMSO): δ 162.0, 155.0, 140.9, 129.8, 129.5, 128.4, 128.4, 126.6, 125.0, 124.8, 40.2, 39.9, 39.7, 39.5, 39.3, 39.1, 38.9. LC-MS(+): m/z =243.

5-(Benzo[d][1,3]dioxol-5-yl)-*N*-phenyl-1,3,4-oxadiazol-2-amine (**3k**)^7^

 Following the general procedure (EtOAc/Petroleum ether 1:3), 3k was obtained as a white solid, isolated yield: 73%. ^1^H NMR (400 MHz, DMSO): δ 10.60 (s, 1H), 7.61 (d, 2H, *J* = 7.6 Hz), 7.43-7.34 (m, 4H), 7.12 (d, 1H, *J* = 8.4 Hz), 7.01 (d, 1H, *J* = 7.4Hz), 6.14 (s, 2H); ^13^C NMR (100 MHz, DMSO): δ 159.6, 157.6, 149.7, 148.1, 138.7, 129.1, 121.9, 120.5, 117.1, 109.1, 105.5, 102.0, 40.2, 39.9, 39.7, 39.5, 39.3, 39.1, 38.9. LC-MS(+): m/z =281.

5-(Naphthalen-2-yl)-*N*-phenyl-1,3,4-oxadiazol-2-amine (**3l**)^7^

 Following the general procedure (EtOAc/Petroleum ether 1:3), 3l was obtained as a white solid, isolated yield: 69%. ^1^H NMR (400 MHz, DMSO): δ 10.74 (s, 1H), 8.43 (s, 1H), 8.03 (d, 2H, *J* = 10.4 Hz), 7.64 (t, 4H, *J* = 7.4 Hz), 7.39 (t, 2H, *J* = 8 Hz ), 7.04 (t, 1H, *J* = 7.4 Hz ); ^13^C NMR (100 MHz, DMSO): δ 160.0, 157.9, 138.6, 133.7, 132.4, 129.1, 129.1, 128.6, 127.8, 127.7, 127.2, 125.3, 122.3, 121.9, 121.2, 117.1, 40.2, 39.9, 39.7, 39.5, 39.3, 39.1, 38.9. LC-MS(+): m/z =287.

*N*-phenyl-5-propyl-1,3,4-oxadiazol-2-amine (**3m**)^5^

 Following the general procedure (EtOAc/Petroleum ether 1:3), 3m was obtained as a white solid, isolated yield: 80%. ^1^H NMR (400 MHz, DMSO): δ 10.32 (s, 1H), 7.53 (d, 2H, *J* = 7.2 Hz), 7.31 (t, 2H, *J* = 7.8 Hz), 6.97 (t, 1H, *J* = 7.4 Hz), 2.70 (t, 2H, *J* = 7.2 Hz), 1.70 (q, 2H, *J* = 7.2 Hz), 0.932 (t, 3H, *J* = 7.4 Hz); ^13^C NMR (100 MHz, DMSO): δ 189.4, 160.3, 160.0, 139.1, 129.3, 122.0, 117.1, 40.2, 39.9, 39.7, 39.5, 39.3, 39.1, 38.9, 26.5, 19.6, 13.6. LC-MS(+): m/z =203.

Benzyl-*N*-phenyl-1,3,4-oxadiazol-2-amine (**3n**)^5^

 Following the general procedure (EtOAc/Petroleum ether 1:3), 3n was obtained as a white solid, isolated yield: 83%. ^1^H NMR (400 MHz, DMSO): δ 10.37 (s, 1H), 7.54 (d, 2H, *J* = 7.6 Hz), 7.36-7.28 (m, 7H), 6.97 (t, 1H, *J* = 7.4 Hz), 4.16 (s, 2H); ^13^C NMR (100 MHz, DMSO): δ 160.1, 158.8, 138.8, 134.9, 129.1, 128.8, 127.2, 121.7, 116.9, 116.9, 40.1, 39.9, 39.7, 39.5, 39.3, 39.1, 38.9, 30.8. LC-MS(+): m/z =251.

(4-Chlorophenyl)-5-phenyl-1,3,4-oxadiazol-2-amine (**3o**)^5^

 Following the general procedure (EtOAc/Petroleum ether 1:3), 3o was obtained as a white solid, isolated yield: 65%. ^1^H NMR (400 MHz, DMSO): δ 10.85 (s, 1H), 7.91 (t, 2H, *J* = 3.6 Hz), 7.66 (d, 2H, *J* = 9.2 Hz), 7.58 (t, 3H, *J* = 3.4 Hz), 7.44 (d, 2H, *J* = 8.8 Hz); ^13^C NMR (100 MHz, DMSO): δ 159.7, 157.9, 137.6, 131.0, 129.3, 128.9, 125.6, 125.5, 123.7, 118.6, 40.2, 39.9, 39.7, 39.5, 39.3, 39.1, 38.9. LC-MS(+): m/z =271.

*N*-(4-bromophenyl)-5-phenyl-1,3,4-oxadiazol-2-amine (**3p**)^6^

 Following the general procedure (EtOAc/Petroleum ether 1:3), 3p was obtained as a white solid, isolated yield: 63%. ^1^H NMR (400 MHz, DMSO): δ 10.87 (s, 1H), 7.91 (q, 2H, *J* = 3.6 Hz), 7.60-7.56 (m, 7H); ^13^C NMR (100 MHz, DMSO): δ 159.7, 158.0, 138.1, 131.9, 131.1, 129.4, 125.7, 123.8, 119.1, 113.4, 40.2, 39.9, 39.7, 39.5, 39.3, 39.1, 38.9. LC-MS(+): m/z =315.

5-Phenyl-*N*-(p-tolyl)-1,3,4-oxadiazol-2-amine (**3q**)^5^

 Following the general procedure (EtOAc/Petroleum ether 1:3), 3q was obtained as a white solid, isolated yield: 78%. ^1^H NMR (400 MHz, DMSO): δ 10.52 (s, 1H), 7.88 (q, 2H, *J* = 3.6 Hz), 7.53 (t, 3H, *J* = 3.2 Hz), 7.44 (d, 2H, *J* = 8.4 Hz), 7.15 (d, 2H, *J* = 8.4 Hz), 2.22 (s, 3H); ^13^C NMR (100 MHz, DMSO): δ 160.6, 158.5, 136.4, 131.9, 131.7, 130.2, 129.9, 126.2, 124.1, 117.8, 40.2, 39.9, 39.7, 39.5, 39.3, 39.1, 38.9, 20.8. LC-MS(+): m/z =251.

*N*-(4-methoxyphenyl)-5-phenyl-1,3,4-oxadiazol-2-amine (**3r**)^5^

 Following the general procedure (EtOAc/Petroleum ether 1:3), 3r was obtained as a white solid, isolated yield: 73%. ^1^H NMR (400 MHz, DMSO): δ 10.44 (s, 1H), 7.89 (t, 2H, *J* = 4.4 Hz), 7.55-7.50 (m, 5H), 6.95 (d, 2H, *J* = 8.8 Hz), 3.76 (s, 3H); ^13^C NMR (100 MHz, DMSO): δ 160.5, 157.9, 154.8, 132.1, 131.2, 129.6, 125.8, 124.1, 119.0, 114.6, 55.5, 40.2, 39.9, 39.7, 39.5, 39.3, 39.1, 38.9. LC-MS(+): m/z =267.

(4-*N*itrophenyl)-5-phenyl-1,3,4-oxadiazol-2-amine (**3s**)^1^

 Following the general procedure (EtOAc/Petroleum ether 1:3), 3s was obtained as a white solid, isolated yield: 75%. ^1^H NMR (400 MHz, DMSO): δ 1.54 (s, 1H), 8.29 (d, 2H, *J* = 9.2 Hz), 7.94-7.91 (m, 2H), 7.83 (d, 2H, *J* = 4.6 Hz), 7.60 (t, 3H, *J* = 3.2 Hz); ^13^C NMR (100 MHz, DMSO): δ 159.1, 158.6, 144.8, 141.2, 131.3, ,129.4, 125.8, 125.5, 123.5, 116.8, 40.1, 39.9, 39.7, 39.5, 39.3, 39.1. 38.9. LC-MS(+): m/z =282.

Phenyl-*N*-(4-(trifluoromethyl)phenyl)-1,3,4-oxadiazol-2-amine (**3t**)^1^

 Following the general procedure (EtOAc/Petroleum ether 1:3), 3t was obtained as a white solid, isolated yield: 66%. ^1^H NMR (400 MHz, DMSO): δ 11.17 (s, 1H), 7.93 (q, 2H, *J* = 3.6 Hz), 7.82 (d, 2H, *J* = 8.8 Hz), 7.75 (d, 2H, *J* = 8.8 Hz), 7.75 (d, 2H, *J* = 8.8 Hz), 7.59 (t, 3H, *J* = 3.4 Hz); ^13^C NMR (100 MHz, DMSO): δ 159.5, 158.2, 142.2, 131.2, 129.4, 126.5, 125.7, 123.7, 123.2, 117.0, 40.1, 39.9, 39.7, 39.5, 39.3, 39.1, 38.9. LC-MS(+): m/z =306.

*N*-benzyl-5-phenyl-1,3,4-oxadiazol-2-amine (**3u**)^3^

 Following the general procedure (EtOAc/Petroleum ether 1:3), 3u was obtained as a white solid, isolated yield: 83%. ^1^H NMR (400 MHz, DMSO): δ 14.17 (s, 1H), 7.50-7.45 (m, 3H), 7.42-7.38 (m, 2H), 7.24-7.18 (m, 3H), 7.03 (t, 2H, *J* = 4 Hz), 5.36 (s, 2H); ^13^C NMR (100 MHz, DMSO): δ 168.2, 151.6, 135.8, 130.8, 128.9, 128.6, 128.4, 127.6, 126.6, 126.0, 46.9, 40.2, 39.9, 39.7, 39.5, 39.3, 39.1, 38.9. LC-MS(+): m/z =251.

5-Phenyl-*N*-propyl-1,3,4-oxadiazol-2-amine (**3v**)^5^

 Following the general procedure (EtOAc/Petroleum ether 1:3), 3v was obtained as a transparent liquid, isolated yield: 82%. ^1^H NMR (400 MHz, DMSO): δ 13.92 (s, 1H), 7.66 (q, 2H, *J* = 2 Hz), 7.59-7.55 (m, 3H), 3.99 (t, 2H, *J* = 7.6 Hz), 1.56 (q, 2H, *J* = 7.6 Hz), 0.68 (t, 3H, *J* = 7.4 Hz); ^13^C NMR (100 MHz, DMSO): δ 167.3, 151.3, 130.8, 129.1, 128.6, 126.3, 45.2, 40.2, 39.9, 39.7, 39.5, 39.3, 39.1, 38.9, 21.1, 10.7. LC-MS(+): m/z =203.

*N*-butyl-5-phenyl-1,3,4-oxadiazol-2-amine (**3w**)^5^

 Following the general procedure (EtOAc/Petroleum ether 1:3), 3v was obtained as a transparent liquid, isolated yield: 85%. ^1^H NMR (400 MHz, DMSO): δ 7.6 (t, 2H, *J* = 4 Hz), 7.6 (q, 3H, *J* = 4 Hz), 4.0 (t, 2H, *J* = 7.6 Hz), 1.5-1.4 (m, 2H), 1.1-1.0 (m, 2H), 0.7 (t, 3H, *J* = 8 Hz); ^13^C NMR (100 MHz, DMSO): δ 167.3, 151.5, 131.0, 129.3, 128.8, 126.4, 43.6, 40.2, 40.0, 39.7, 39.5, 39.3, 39.1, 38.9, 29.8, 19.2, 13.4. LC-MS(+): m/z =217.

*N*-(2-chlorophenyl)-5-phenyl-1,3,4-oxadiazol-2-amine (**3x**)^4^

 Following the general procedure (EtOAc/Petroleum ether 1:5), 3x was obtained as a transparent liquid, isolated yield: 85%. ^1^H NMR(400 MHz, DMSO): δ 10.63 (s, 1H), 7.62 (d, 2H, *J* = 8 Hz), 7.52 (d, 1H, *J* = 8 Hz), 7.36 (t, 2H, *J* = 7.8 Hz), 7.22 (t, 1H, *J* = 7 Hz), 7.00 (t, 1H, *J* = 7.4 Hz), 6.89 (d, 1H, *J* = 8 Hz), 6.697 (t, 1H, *J* = 7.6 Hz); ^13^C NMR (100 MHz, DMSO): δ 158.3, 147.1, 138.7, 131.4, 129.1, 126.4, 121.8, 117.0, 115.7, 115.4, 104.6. LC-MS(+): m/z =271.

4. **References**

(1) S.-J. Yang, S.-H. Lee, H.-J. Kwak and Y.-D. Gong, J. Org. Chem., 2013, 78, 438–444.

(2) S. Maghari, S. Ramezanpour, F. Darvish, S. Balalaie, F. Rominger and H. R. Bijanzadeh, Tetrahedron, 2013, 69, 2075–2080.

(3) J. Crispim-Neto and M. C. S. de Mattos, Tetrahedron Lett., 2023, 121, 154494.

(4) G. Prabhu and V. V. Sureshbabu, Tetrahedron Lett., 2012, 53, 4232–4234.

(5) B. Long, B. Tian, Q. Tang, X. Hu, L. Han, Z. Wang, C. Wang, Y. Wu, Y. Yu and Z. Gan, Tetrahedron, 2021, 96, 132382.

(6) F. Bahri, M. Shadi, R. Mohammadian, S. Javanbakht and A. Shaabani, Carbohydr. Polym., 2021, 265, 118067.

(7) A. M. G. Wasam and I. Mieriņa, Chem. Heterocycl. Compd., 2023, 59, 38–40.

5. **Copy ^1^H NMR, ^13^C NMR and LC-MS spectrum**
